# Botanical Flavonoids: Efficacy, Absorption, Metabolism and Advanced Pharmaceutical Technology for Improving Bioavailability

**DOI:** 10.3390/molecules30051184

**Published:** 2025-03-06

**Authors:** Lei Hu, Yiqing Luo, Jiaxin Yang, Chunsong Cheng

**Affiliations:** 1 Jiangxi Key Laboratory for Sustainable Utilization of Chinese Materia Medica Resources, Lushan Botanical Garden, Chinese Academy of Sciences, Jiujiang 332900, China; hulei2021cm@163.com (L.H.); 18618405253@163.com (Y.L.); yangjx@lsbg.cn (J.Y.); 2 Lushan Xinglin Institute for Medicinal Plants, Jiujiang Xinglin Key Laboratory for Traditional Chinese Medicines, Jiujiang 332900, China

**Keywords:** flavonoids, efficacy, in vivo study, bioavailability, modification

## Abstract

Flavonoids represent a class of natural plant secondary metabolites with multiple activities including antioxidant, antitumor, anti-inflammatory, and antimicrobial properties. However, due to their structural characteristics, they often exhibit low bioavailability in vivo. In this review, we focus on the in vivo study of flavonoids, particularly the effects of gut microbiome on flavonoids, including common modifications such as methylation, acetylation, and dehydroxylation, etc. These modifications aim to change the structural characteristics of the original substances to enhance absorption and bioavailability. In order to improve the bioavailability of flavonoids, we discuss two feasible methods, namely dosage form modification and chemical modification, and hope that these approaches will offer new insights into the application of flavonoids for human health. In this article, we also introduce the types, plant sources, and efficacy of flavonoids. In conclusion, this is a comprehensive review on how to improve the bioavailability of flavonoids.

## 1. Introduction

Flavonoids are a class of natural polyphenolic compounds widely found in plants and are part of the secondary metabolites of plants [1]. They are mainly present in various parts of fruits, vegetables, tea leaves, nuts, flowers and other plants. Citrus fruits (such as oranges and lemons), grapes, apples, and berries are rich in flavonoids, including hesperidin, anthocyanins, and quercetin. Green leafy vegetables such as spinach, kale, onions, and broccoli are also important sources of flavonoids [2]. In addition, theaflavins and catechins in tea, quercetin in nuts, and isoflavones in soybeans (such as soy isoflavones) are typical sources of flavonoids [3,4].

Flavonoids in nature connect to sugar molecules (glycosyl) through glycosidic linkage to form flavonoid glycosides. Aglycones are non-sugar parts of glycosides, for example, quercitrin (quercetin-3-O-glucoside) are glycosides, while quercetin are aglycones. The activities of flavonoid glycosides and their aglycones are quite different. In general, flavonoid glycosides exhibit greater hydrophilicity compared to their corresponding aglycones, while they tend to have lower lipophilicity than the aglycones. Flavonoids (aglycones) exhibit high bioactivity, but their stability is very poor. When the sugar substituent binds to the aglycones through glycosidic linkage, the bioactivity of the flavonoids decreases but their stability is improved [5]. In terms of drug activity, flavonoid glycosides may be better than their aglycones [6]. In addition, glycosylation can increase the hydrophilicity of flavonoid aglycones, reduce toxic side effects, and improve the specific targeting ability of the drug [6]. In terms of bioavailability, flavonoid glycosides are generally lower than those of aglycones [7]. But this phenomenon is not absolute, because the plasma concentration of flavonoid-C glycosides seems to be significantly higher in the body than their aglycones [8]. Furthermore, glycosylation is also believed to protect plants from self-oxidative damage, although the biological activity of flavonoids may decrease [8].

As natural products, flavonoids have various positive effects on plants. These compounds are the natural coloring agents of plants, especially anthocyanins, which are important players in plants’ ability to display their vibrant colors [9]. At the same time, the dazzling colors that are made possible by the presence of flavonoids can attract animals that spread seeds on behalf of the plant itself [10]. Flavonoids are also associated with plant growth and development and play a role in the growth of plant leaves through their antioxidant capacity and regulatory properties [11]. Of course, as essential players in nature, not all plants are at the top of the pyramid. Most face numerous biotic or abiotic stresses, such as being “tasty meals” for herbivores, invasive pathogens, UV radiation, drought, and more. It seems that flavonoids have become the main means by which plants combat these stresses [12]. Flavonoids play a significant role in protecting plants from herbivores by altering their taste, reducing their digestibility, and increasing toxicity at high concentrations [13]. Flavonoids have also been found to combat fungal or bacterial infections in plants by inducing apoptosis and mitochondrial dysfunction, as well as by blocking cell wall synthesis [14,15]. Another example is the finding that flavonoids from maize species enhance the plant’s drought tolerance by regulating drought-induced oxidative damage and stomatal movement [16].

Meanwhile, they also have positive effects on human health through dietary intake, including antioxidant, antitumor, anti-inflammatory, antimicrobial and other bioactivities [2]. Although increasing studies have shown the great potential of flavonoids in promoting human health, the limiting factors of their bioavailability are often overlooked when explaining their “human-friendly” bioactivities. In other words, certain flavonoids can indeed promote human health, but the questions of how, how much, and how effectively the body can use flavonoids are often understated. In fact, the bioavailability of flavonoids is relatively low, mainly due to the following factors. First, the interaction between flavonoids and other nutrients, for example, fat intake will increase its bioavailability, while protein intake will reduce its bioavailability [17]; the second is the metabolic behavior of the liver (Phase I and Phase II), such as the methylation, sulfation or glucuronidation of flavonoids [18]; the third is the interaction with the gut microbiome. It is worth noting that after being “processed” by microorganisms, the bioactivity of some flavonoids may even improve [18]. Additionally, factors such as diet, genetics, and metabolic diseases can significantly affect flavonoid bioavailability, influencing how effectively these compounds are absorbed and utilized by the body. In short, when considering the bioactivity of flavonoids, their bioavailability must be taken into account first, because their bioactivity depends largely on their bioavailability.

Research on the structural characteristics, bioactivities and in vivo processes of flavonoids is the key point for trying to improve their bioavailability. Modern science and technology have enabled the modification of drugs or compounds through formulation technology or chemical modification methods, allowing certain physical and chemical properties of the original compound to be altered towards an ideal state. In this review, we primarily discuss how to improve the bioavailability of flavonoids and propose that it can be achieved by changing the dosage form of the drug and through chemical modification, providing a theoretical basis for the long-term development of flavonoids in the field of medical health.

In order to complete this manuscript, we performed an exhaustive and methodical review of the literature by querying several prominent scientific databases, including PubMed, ScienceDirect, Web of Science, Scopus, Google Scholar, SpringerLink, Wiley Online Library. The search was conducted using a combination of key terms, such as flavonoids, biological activity, plant sources, gut microbiome, nano preparation, and structural modifications. This comprehensive search strategy facilitated the acquisition of the most relevant and current research, ensuring a thorough and up-to-date understanding of the biological properties, health-promoting potential, and applications of flavonoids. Furthermore, particular attention was given to the exploration of plant-based sources, the interaction between gut microbes and flavonoid metabolism, and the latest advancements in pharmaceutical technologies, which collectively provided a well-rounded and nuanced perspective on the subject matter.

## 2. Classification of Flavonoids

**Figure 1:**
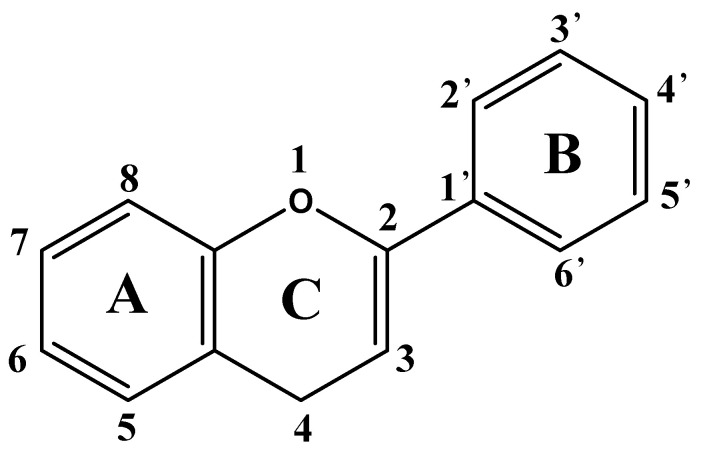
Basic skeleton and active sites of flavonoids.

Flavonoids are a class of compounds with diphenyl chromone as the basic nucleus. They are C6–C3–C6 structural substances consisting of two benzene rings (A and B rings) and one pyran ring (C ring, a three-carbon structure) [19] (Figure 1).

**Figure 2:**
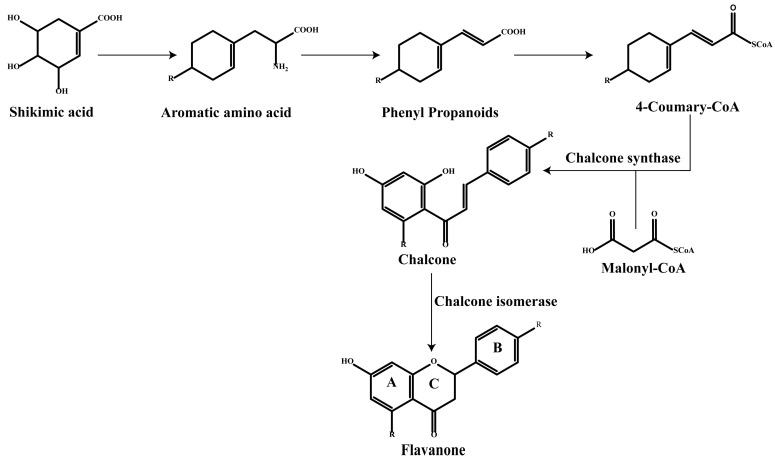
The synthetic pathway of flavonoids.

It is worth noting that there are two ways for plants to synthesize flavonoids, one is the shikimic acid pathway, which produces a phenylpropanoid (C6–C3) skeleton, and the other is the acetate pathway, which provides building blocks for polymerizing two-carbon units [1]. The acetate pathway generates ring A, the shikimic acid pathway generates ring B, ring A and ring B condense to form chalcone (a precursor of flavonoids), which then undergoes catalysis by chalcone isomerase to form the heterocyclic (C-ring) flavanone. And flavanone is the starting compound for the synthesis of other flavonoids [1,20] (Figure 2).

To date, more than 10,000 natural flavonoids have been identified [21]. According to the connection point between the benzene ring (B ring) and the pyran ring, and whether the central three-carbon chain is cyclized and the degree of oxidation, flavonoids can be roughly divided into the following subcategories: flavones, isoflavones, flavonols, flavanols, flavanones chalcones, and anthocyanidins [22] (Table 1). Catechins are the most abundant flavanols in tea. While flavanones are mainly derived from citrus fruits, among which hesperidin, naringenin and paclitaxel are the most representative. Flavones such as luteolin and apigenin can be found in celery, tea, red pepper, and oranges [22]. The main sources of isoflavones are beans and their derivatives, with genistein and glycitein being the representative compounds. Quercetin is the most widely studied flavonol compound, mainly derived from onions, kale, leeks, and other dietary sources. Chalcone is a natural open-chain flavonoid and a precursor to all other flavonoids [23]. Xanthohumol and Corylifolinin are the most representative chalcone compounds owing to their rich pharmacological activities. Anthocyanidins are natural dyes found in plants, giving flowers and fruits their attractive colors [24]. The most abundant anthocyanidins include delphinidin, cyanidin, petunidin, peonydin, malvidin, and pelargonidin [25].

## 3. Efficacy of Flavonoids

### 3.1. Antioxidant

In 1985, a monograph titled “Oxidative Stress” first introduced the concept of oxidative stress, in which oxidative stress is described as oxidative damage to cells and organs [47]. Oxidative stress generally refers to the phenomenon in which the balance between oxidation and anti-oxidation is disrupted or reactive oxygen species (ROS)/reactive nitrogen species (RNS) are excessively produced in organisms to induce inflammatory infiltration of neutrophils and trigger a series of cellular damage and pathological reactions. ROS include superoxide anion, hydroxyl radical, hydrogen peroxide and singlet oxygen; RNS include nitric oxide, nitrogen dioxide and peroxynitrite [48]. Correspondingly, according to the nature of antioxidants, the system that antagonizes oxidative stress in the body can be roughly divided into enzymatic and non-enzymatic systems. The enzymatic antioxidant system includes superoxide dismutase (SOD), catalase (CAT) and glutathione peroxidase (GSH-Px). The non-enzymatic antioxidant system includes vitamin C, vitamin E, carotenoids and some trace elements such as selenium and zinc. Some views in modern molecular biology suggest that oxidative stress is the result of the disruption of the thiol redox pathway (controlled by thioredoxin Trx, glutathione GSH and cystedin Cys). The influence of the thiol redox pathway on some pathways, such as HRas, PTP-18, Nrf2, NF-κB, etc., can significantly interfere with normal physiological activities of the human body [49,50].

Flavonoid compounds can directly remove excessive ROS in the body due to their special structural morphology. The specific activity intensity is arranged in the following order: Flavanol (Flavan-3-ols) (4.21 mM) > Flavonol (1.83 mM) > Chalcone (1.51 mM) > Flavonoid (0.91 mM) > Flavanone (0.16 mM) > Isoflavone (0.08 mM) [51]. In short, the activity of flavonoids in scavenging ROS depends on their own structure, the number and arrangement of hydroxyl groups play an important role in the antioxidant effect of flavonoids [52,53]. This effect may be attributed to the hydrogen atoms provided by the hydroxyl structure or the electron delocalization effect triggered by conjugation with the oxygen group, which leads to the scavenging of free radicals [51], and the hydroxyl structure of the B ring is the most important factor in determining the clearance of ROS and RNS compared to the A and C rings [54].

Flavonoids can also play an antioxidant role by activating antioxidant enzymes. Studies have shown that flavonoids activate electrophile-responsive element (EpRE) to regulate multiple phase II detoxification enzymes including NAD(P)H quinone oxidoreductase (NQO), glutathione S-transferase (GST), and UDP glucuronyl transferase (UGT), thereby resisting oxidative stress [54]. Correspondingly, these substances can also exert their antioxidant stress resistance by inhibiting oxidases. Currently, flavonoid compounds have been shown to inhibit cyclooxygenase, lipoxygenase, microsomal monooxygenase, nicotinamide adenine dinucleotide (NADH) oxidase, xanthine oxidase, and others [22]. Studies have also shown that quercetin can inhibit the burst of oxidative stress by downregulating the protein kinase C (PKC) pathway to block its phosphorylation process of nicotinamide adenine dinucleotide phosphate (NADPH) oxidase (NOX) subunit p47phox [55].

In addition, flavonoids are also considered to form chelates with metal ions to combat oxidative stress. One study pointed out that quercetin and morin exert antioxidant effects by antagonizing the decrease in the activities of SOD and GSH induced by cadmium (Cd). Compared with the Cd-only group, the SOD and GSH activities in mice receiving quercetin + Cd or mulberry + Cd groups showed significantly higher activity (*p* ⩽ 0.001) [56].

Oxidation of human low-density lipoprotein (LDL) is associated with atherosclerosis, and α-tocopherol is an antioxidant for LDL. Studies have shown that flavonoids such as quercetin, epigallocatechin, epigallocatechin gallate, epicatechin, epicatechin gallate and naringin can act as hydrogen donors to reduce α-tocopherol free radicals or promote the production of α-tocopherol, thereby delaying the oxidation of LDL [57,58].

Of course, there are some relatively new views suggesting that flavonoid compounds play an antioxidant role by increasing the level of uric acid in plasma, but this view still lacks specific data support [59]. Some studies have also shown that when β-carotene is combined with flavonoid compounds such as hesperidin, rutin, and quercetin, it exhibits an anti-DNA-damage effect under UV-A irradiation [60].

In summary, the mechanisms of flavonoids against oxidative stress can be summarized as follows:

1. The hydroxyl structure of flavonoids acts as a hydrogen atom donor to neutralize free radicals to directly remove ROS.
2. The oxo group of flavonoids participates in the conjugated system to enhance electron delocalization.
3. Activation of antioxidant enzymes activity.
4. Inhibition of oxidase activity.
5. Formation of metal chelates.

Finally, it is worth noting that under pathological conditions, high levels of free radicals can damage molecules such as nucleic acids, proteins, and lipids, ultimately leading to cell aging and death, as well as promoting the development of tumors [61,62,63]. Therefore, the antioxidant biological properties of flavonoids may be the prerequisite for their other pharmacological activities, especially antitumor activity [64].

### 3.2. Anticancer

Uncontrolled abnormal proliferation of normal cells in the body is called cancer [65]. Various cellular activities such as growth, proliferation, apoptosis, and metastasis of tumor cells are regulated by multiple important signal cascades such as Nuclear factor erythroid 2-related factor 2 (Nrf2), Cyclic GMP-AMP synthase-stimulator of interferon genes (cGAS-STING), WNT/β-catenin, Rat Sarcoma/Rapidly Accelerated Fibrosarcoma/Mitogen-Activated Protein Kinase/Extracellular Signal-Regulated Kinase (Ras/Raf/MEK/ERK), Epidermal growth factor receptor (EGFR), Phosphoinositide-specific Phospholipase C/Protein kinase C (PLC/PKC), Phosphoinositide 3-Kinase/Protein kinase B (PI3K/AKT), Notch Receptor (NOTCH), Platelet-derived growth factor (PDGF), Vascular Endothelial Growth Factors (VEGF), Cellular-mesenchymal epithelial transition factor (c-MET), Yes-associated protein/Transcriptional Enhanced Associate Domains (YAP/TEAD), Phospho P38 mitogen-activated protein kinase/Mitogen-activated protein kinase/Extracellular regulated protein kinases (P38/MAPK/Erk), Toll-like receptors (TLR) etc.) [66,67,68,69,70,71,72,73,74,75]. It is worth noting that these pathways are not involved in regulation in isolation. Pathways are closely related to each other to form signaling cascades. Usually, inhibition of such signaling pathways/cascades can significantly inhibit the development of cancer and even overcome the problem of drug resistance [76,77,78].

Currently, a large number of studies have shown that flavonoid compounds can exert a powerful tumor-inhibitory effect, including the regulation of multi-level signaling pathways, key proteins, and factors [79]. Take Taxifolin (TAX) as an example: TAX is a flavonoid derivative found in natural plants. Studies have shown that TAX exerts tumor suppression by regulating the MAPK signaling pathway. Specifically, the regulation of the MAPK pathway includes the inhibition of pERK, pJNK, p-p38, Rac1, and c-Myc. This result is supported by Western blotting experiments [80]. The study also found that TAX inhibited the nuclear translocation of the tumor cell division gene β-catenin by inhibiting Rac1/JNK signaling. The expression of the β-catenin downstream gene, microphthalmia transcription factor (MITF) was inhibited in B16F10 and A375 cell lines treated with TAX [80]. Research by Manigandan et al. [81] revealed a potential mechanism for TAX in fighting rectal cancer. TAX stimulates the expression of the Nrf2 protein by inhibiting Wnt/β-catenin signaling, while Nrf2 protects cells from oxidative stress and DNA damage by enhancing the expression of antioxidant enzymes, which is believed to reduce the risk of cancer development. However, excessive activation of Nrf2 may promote the malignant proliferation of cancer cells, enhance the invasiveness and metastasis of tumor cells, and induce drug resistance. Therefore, it remains to be determined whether TAX-induced Nrf2 expression also plays a positive role in other types of cancer besides rectal cancer [82]. A previous study found that both the flavonoid biochanin A and the chemotherapy drug temozolomide inhibited the cell viability of human glioblastoma U87 MG cells in a dose-dependent manner. Compared to the control group, the inhibitory effect of combined administration of the two was more significant (*p* ≤ 0.05), subsequent Western blotting experiments confirmed the specific inhibitory mechanism. The experiment found that the combined drug treatment inhibited the levels of p-ERK, p-AKT, EGFR and c-myc, and upregulated the expression of phosphorylated p53 (p-p53) [83]. In addition, the inhibition of flavonoids such as hesperidin, naringin, quercetin, luteolin, and apigenin on NF-κB, ERK1/2, AP1, TNF-α, PKC, and Nrf2 pathways has been proved to have significant pharmacological activities on a variety of cancers, including hepatocellular carcinoma, colon cancer, breast cancer, lung cancer, cervical cancer, and bladder cancer. Of course, these activities may be attributed to the antioxidant activity of flavonoids [64]. The issue of tumor resistance has long been a hot topic of research. Recently, some scholars have pointed out that flavonoids and their conjugates interact with efflux proteins (mainly the ABC transporter family), especially P-glycoprotein and breast cancer resistance protein (BCRP). The interaction between flavonoids and these ABC transporters (ATP binding cassettes) may be an important way to enhance the bioavailability of chemotherapeutic drugs and overcome tumor resistance [78].

### 3.3. Anti-Inflammatory

Inflammation is a complex pathological and physiological process associated with a variety of diseases, involving the activation of both immune and non-immune cells [84]. Through the inflammatory response, invading pathogens or dysfunctional cells are eliminated to promote tissue repair and restoration of homeostasis [85]. Flavonoids have been shown to have strong anti-inflammatory effects and can inhibit the development of inflammation by inhibiting the production of inflammatory mediators such as prostaglandins and leukotrienes. Quercetin is a flavonoid with multiple physiological activities, including antitumor and anti-inflammatory effects. A recent study found that quercetin inhibited the expression of cyclooxygenase-2 (COX-2) protein and COX-2 promoter activity in MDA-MB-231 cells in a dose-dependent manner [86]. Nobiletin was found to block the mRNA expression of inducible nitric oxide synthase (iNOS) and COX-2 and significantly inhibited LPS-induced nitric oxide (NO) and prostaglandin E2 (PGE2) production [87].

In addition, flavonoids inhibit the progression of inflammation by regulating the nuclear factor κB (NF-κB) signaling pathway [88,89,90]. NF-κB is an important transcription factor, involved in the regulation of various inflammatory reactions [91]. During an inflammatory response, NF-κB promotes the release of pro-inflammatory factors such as TNF-α, IL-1β and IL-6 by activating the transcription of downstream genes [92]. Flavonoids can modulate the NF-κB pathway through multiple mechanisms, thereby reducing the production of pro-inflammatory factors. Green tea polyphenols have been found to alleviate silicosis by inhibiting the expression of proinflammatory factors TNF-α, IL-1β, and IL-6 by inhibiting the IL-17/NF-κBp65 signaling cascade [93]. Other studies have shown that flavonoids can bind to IκB kinase (IKK) and inhibit the nuclear translocation of NF-κB, thereby significantly reducing inflammatory responses [94,95].

It is worth noting that flavonoids can also play an anti-inflammatory role as antioxidants. Flavonoids inhibit inflammatory factors or promote the expression of antioxidant genes by regulating classic inflammatory response pathways (such as TLR4-NF-κB, PI3K-AKT and Nrf2/HO-1 signaling pathways), thus playing an anti-inflammatory role in the cardiovascular system [96].

### 3.4. Antimicrobial

In recent years, as the problem of antibiotic resistance has become increasingly serious [97], researchers have paid more and more attention to the potential of flavonoids as natural antimicrobial agents. Flavonoids can combat bacterial infection through many mechanisms, including inhibiting bacterial cell wall synthesis, damaging bacterial cell membranes, inducing oxidative stress, inhibiting protein synthesis, and interfering with bacterial DNA replication and transcription [98]. A recent study explained the antibacterial mechanism of the flavonoids sophoraflavanone G and kurarinone. The results showed that these two substances inhibited the bioactivity of *Staphylococcus aureus* in a dose-dependent manner, and even their MIC_90_ (the minimum concentration of the tested drug that inhibits 90% bacterial growth) remained stable after 20 consecutive subcultures. This indicates that *Staphylococcus aureus* has a higher sensitivity to these two substances. Further studies have found that these two substances mainly exert their corresponding antibacterial effects by targeting the cell membrane of *Staphylococcus aureus*, causing structural damage to the membrane, and inhibiting the biosynthesis of the cell membrane and cell wall [99]. Pyocyanin and elastase are important virulence factors of *Pseudomonas aeruginosa*, which are related to its biofilm formation and immune evasion, respectively [100,101]. Naringenin was found to exert its antibacterial properties by inhibiting both pyocyanin and elastase [102]. In addition, some studies have shown that flavonoids such as epigallocatechin gallate (EGCG) and theaflavin-3,3′-digallate (TF3) exert anti-Clostridium perfringens effects in a concentration-dependent manner by inhibiting proteins involved in septum formation, DNA separation, and cell division [103]. It is worth noting that Lee et al. revealed an unusual antibacterial mechanism of flavonoids for the first time. In this study, it was found that the flavonoid propolin D from *Macaranga tanarius* (L.) inhibited the formation of Candida albicans biofilm by reducing the formation of hyphae and inhibited the formation of *Enterohemorrhagic Escherichia coli* (EHEC) biofilm by reducing the formation of curly fimbriae. The results were demonstrated by qRT-PCR. These results showed that propolin D could significantly downregulate the gene expression of ECE1, HWP1, and curli subunits (csgA and csgB) to inhibit the formation of biofilm [104].

## 4. Pharmacokinetics of Flavonoids

Most flavonoids exist in glycoside form (except flavanol) [105]. Flavonoids are absorbed and metabolized in the form of glycosides or aglycones, but the specific mechanisms underlying the difference in absorption ability between glycosides and aglycones are still unknown [18]. Usually, these compounds are absorbed in the small intestine (except flavan-3-ols and proanthocyanidins) [106]. The absorption mechanism was divided into two types based on the cleavage enzymes present in the brush border epithelial cells of the small intestine. One mechanism is thought to be the conversion of flavonoids to their aglycone forms by the enzyme lactase phlorizin hydrolase (LPH), which, due to their increased lipid solubility, allows them to enter the intestinal epithelial cells via passive diffusion. The second mechanism involves the transfer of hydrophilic glycosides by certain membrane transporters, such as sodium-dependent glucose transporter 1 (SGLT-1) and glucose transporter 2 (GLUT-2) [107]. After transport by SGLT-1 or GLUT-2, cytosolic β-glucosidase (CBG) or LPH hydrolyzes flavonoid glycosides into their aglycones for absorption [108,109]. Both aglycones and their corresponding glycosides can be absorbed through these two pathways, but in comparison, the absorption efficiency of flavonoid aglycones is higher than that of their corresponding glycosides. In a Caco-2BBe1 cell model, it was found that the metabolic kinetics of quercetin and anthocyanins differ significantly from their respective monoglycosides or bisglycosides. High levels of aglycone substances rather than glycoside substances were detected in cells and on the basolateral side. This suggested that aglycone substances have higher cellular uptake capacity and transport efficiency. However, when glycone groups are introduced into the aglycone structure, lower cellular uptake and transport behavior can be observed, and the greater the number of glycone groups, the lower the uptake and transport efficiency [107].

After the administration of quercetin or quercitrin, it is difficult to detect the components of these two substances in plasma, but their glucuronides, sulfates or methyl conjugates can be detected, which reminds us that some unexpected changes may occur in flavonoids after oral administration [110]. Studies have shown that such substances undergo hepatic metabolism by phase I hydroxylation of the aromatic ring and phase II metabolism by binding to UPD-glucuronosyltransferase (Glucuronidation), glutathione S-transferase (sulfation), and catechol-O-methyltransferase (methylation) after ingestion [111]. Glucuronidation and sulfation aim to increase the water solubility of flavonoids for renal excretion. The ortho-hydroxyl groups on the structure of flavonoids are methylated by catechol-O-methyltransferase, and the methylated flavonoids are primarily excreted through the kidneys [111]. In summary, glucuronidation, sulfation, and methylation serve to enhance the water solubility of these substances, promoting their excretion through bile and urine [105]. Furthermore, this process is also a key mechanism that limits the toxic potential of flavonoids [105]. Of course, some very interesting phenomena were revealed during the research process. Flavonoids seem to be more inclined to undergo sulfation rather than glucuronidation, but they can also transform to glucuronidation after sulfation. Of course, such a transformation needs to be established under the condition of large-scale intake [112]. The balance between sulfation and glucuronidation also seems to be affected by a number of factors, such as gender, species, and whether or not the individual is fasting [113].

**Figure 3:**
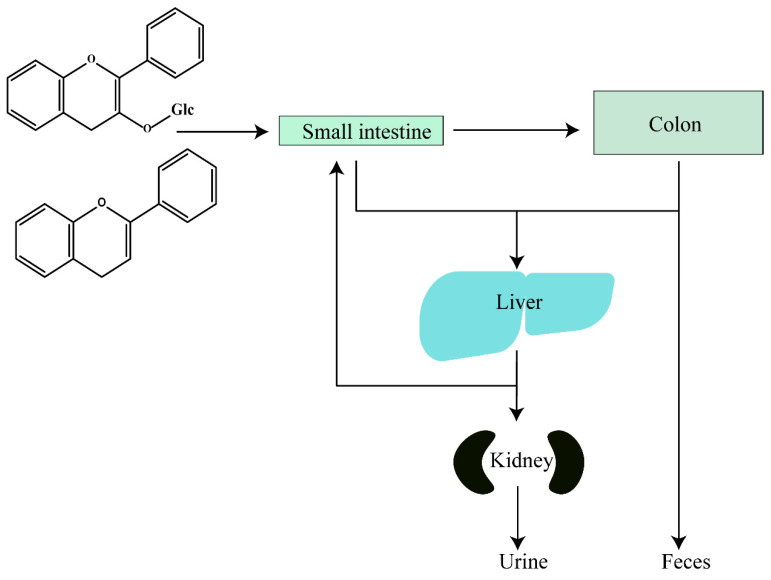
Metabolic pathways of flavonoids.

Notably, flavonoids that have been absorbed by the small intestine enter the liver through the portal vein and are metabolized by hepatic enzymes and released into circulation, increasing the amount of polar conjugates excreted in the urine or returned to the duodenum through the gallbladder [22,114]. Meanwhile, most unabsorbed flavonoids enter the large intestine, where abundant gut microbiomes cleave their pyranone rings, undergo dehydroxylation, decarboxylation, and other reactions, further metabolizing the flavonoids, which are subsequently absorbed or excreted in the feces [115,116] (Figure 3). Of course, gut microbiomes may also change the structure of flavonoids to enhance their bioactivity and improve their bioavailability. But interestingly, some compounds that have been metabolized in the blood may even re-enter the liver circulation for further metabolism and eventually be excreted from the body in urine [108].

## 5. Flavonoids and Gut Microbiome

**Figure 4:**
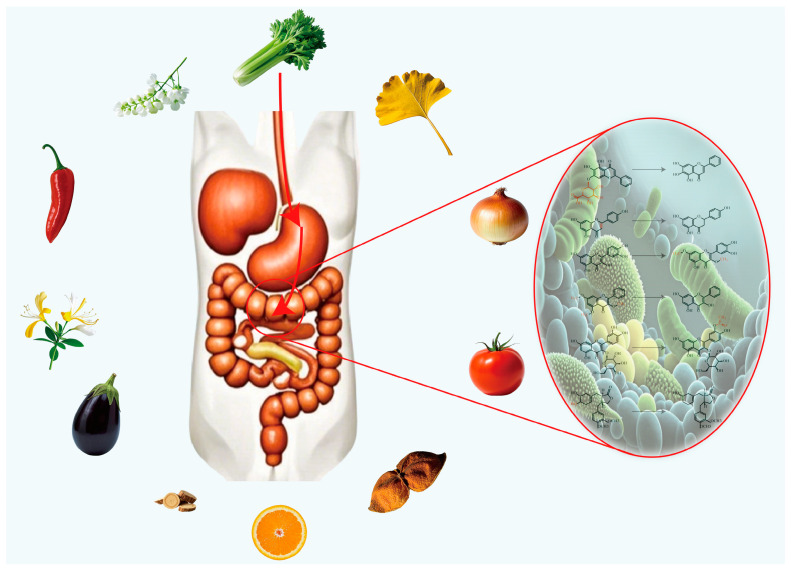
Processing of intestinal microbiome.

Some studies have suggested that the interaction between the gut microbiome and flavonoids may be a key factor in promoting the beneficial effects of flavonoids. That is, the gut microbiome may affect the utilization or biotransformation of flavonoids, and flavonoids may also affect the growth of microorganisms present in the intestine [117]. We may hypothesize that if the population and number of microorganisms in the intestinal tract that are “friendly” to flavonoids change, then the flavonoids ingested orally may lose most of their physiological activities that are beneficial to the human body, and this is a concern that requires vigilance. Previous studies have found that there are a large number of microorganisms in the intestine (about 10^5^ in the small intestine, about 10^3^ to 10^7^ in the ileum, and about 10^12^ in the colon) [118]. These microorganisms will produce a large number of enzymes during growth and metabolism. Under the action of these enzymes, the flavonoids entering the intestinal tract will undergo reactions such as hydrolysis, reduction, deketolization, decarboxylation, etc. generating new microbial metabolites (Figure 4). These “processed” flavonoids undergo structural changes, which contribute to alterations in their bioactivity and bioavailability [119,120]. For example, flavonoid aglycones with 5-hydroxyl groups in their structure can be degraded faster in the intestine than structures without 5-hydroxyl groups [121], and structures without methoxy groups can be degraded faster than structures with methoxy groups [122].

### 5.1. Hydrolysis Reaction

In nature, flavonoids mainly exist in the form of glycosides combined with glycone groups. The glycosidic bond between the flavonoid and the glycone groups dissociates, resulting in the release of the flavonoid aglycone [123]. A group of active ingredients metabolically produced by gut microbiome and referred to as fecal microbial enzymes, such as α-rhamnosidase, β-glucosidase and β-glucuronidase, have been shown to be major players in the hydrolytic reactions of flavonoid glycosides in the intestinal tract. Higher levels of β-glucosidase and β-glucuronidase were observed in a diarrhea piglet model, both enzymes are derived from *Escherichia* spp. The increase in the number of *Escherichia* spp. under diarrheal conditions promoted the activity of these two enzymes. In subsequent experiments, it was also found that β-glucosidase and β-glucuronidase promoted the metabolism of daidzin, baicalin and wogonoside etc., because their corresponding daidzein, baicalein and wogonin etc. were detected in the body, and flavonoids in the form of aglycones seemed to have better therapeutic effects in fighting diarrhea [124]. Previously, a strain called PUE was found to convert [6″, 6″-D2] puerarin containing C-glucose into daidzein and [6″, 6″-D2] glucose (labeled with deuterium), the process believed to be caused by C-1 hydroxylation hydrolysis triggered by PUE” [125]. A study conducted some time ago found that the Bacteroides distasonis strain was present in the feces provided by volunteers, and this strain participated in the hydrolysis of flavonoids by providing α-rhamnosidase and β-galactosidase. It is reported that under the action of these two hydrolases, quercitrin and rutin were hydrolyzed to quercetin, and robinin was further hydrolyzed to kaempferol [126]. In general, based on previous learning experience, there are several rules regarding the difficulty of hydrolysis of flavonoid glycosides:

1. Depending on the type of glycoside bond, flavonoid glycosides undergo hydrolysis in roughly the following order of difficulty C-glycosides, S-glycosides, O-glycosides, and N-glycosides.
2. Among pyranosides, the hydrolysis rates are ranked from fast to slow as follows: pentose, methylpentose, hexose, heptose, glucuronide.
3. Amino-glycosides are more difficult to hydrolyze than hydroxy-glycosides, which are more difficult to hydrolyze than deoxyglycosides: 2-aminoglycosides < 2-hydroxyglycosides < 3-deoxyglycosides < 2-deoxyglycosides < 2, 3-deoxyglycosides.
4. Ketoglycosides are more easily hydrolyzed than aldosides.
5. Aromatic glycosides are more easily hydrolyzed than aliphatic glycosides.
6. Furanosides are more easily hydrolyzed than pyranosides.

### 5.2. Reduction Reaction

There are a large number of anaerobic bacteria in the gastrointestinal tract [127], and the double bond between C2 and C3 of flavonoids is easily hydrogenated and reduced [128]. A recent study attracted attention, reporting a previously undiscovered a distinct class of ene-reductases called flavonoid reductase (FLR). The researchers evaluated the metabolites of apigenin by co-culturing it with *Escherichia coli* expressing the A4U99_05915 gene (encoding the KGF53654.1 protein). As a result, the consumption of apigenin and the production of naringenin were observed in the supernatant. Further studies have shown that the FLR encoded by the A4U99_05915 gene has extremely high catalytic properties for flavonoids and flavonols, and it is catalytically active only against these two types of substrates and can catalyze the hydrogenation of the C2 = C3 double bond on the C ring of flavonoids and flavonols, generating dihydroflavones and dihydroflavonols [129]. In addition, flavonoids can also undergo a reduction reaction between the C2-O bonds under the action of the gut microbiome, resulting in the cleavage of the C ring. After ring opening, the O-position and C3-position of some flavonoids, such as flavonols, will recombine to form a five-membered ring [129,130]. There are also some more complex reactions, such as the opening of the C2 and O positions of apigenin under the action of *E.ramulus* leading to the formation of phloretin (chalcone). And the chalcone is further reduced to form dihydrochalcone, which ultimately produces phloroglucinol and p-hydroxyphenylpropionic acid [131].

### 5.3. (De)Methylation, Acetylation, Dehydroxylation Reactions

Flavone glycosides are hydrolyzed to produce their aglycone part (flavonoid parent core). In addition to being partially absorbed by the small intestine, the unused flavonoid parent core structure is prone to methylation, demethylation, acetylation, and dehydroxylation in the small intestine. It has been reported that after methylation, the bioactivity of flavonoids will be greatly improved [132,133]. The flavonoid O-methyltransferase (SaOMT-2) has been cloned from *Streptomyces avermitilis* and has been found to have a wide range of substrate properties, capable of methylation of various flavonoids, including isoflavones, flavonoids, flavonols, and flavanones [134]. Of course, flavonoids will undergo demethylation reactions under the influence of certain intestinal flora. For example, the intestinal bacterium *Blautia* sp. MRG-PMF1 produces a methyltransferase that catalyzes the complete demethylation of 5,7-dimethoxyflavone (5,7-DMF), 3,5,7-trimethoxyflavone (3,5,7-TMF), 3,5,7,3′, 4′-pentamethoxyflavone and other substances to produce their corresponding demethylation products [135]. In addition to methylation, acetylation is another common modification by intestinal bacteria. The main metabolites of hyperoside in the intestine were identified using ultra-high-performance liquid chromatography/quadrupole time-of-flight mass spectrometry. The results showed that in the presence of intestinal bacteria hyperoside was metabolized into its corresponding acetylated products and dehydroxylated products [136]. Some bacteria can also catalyze the dehydroxylation of flavonoids. The dehydroxylation reaction is unique in the biotransformation between catechins and gut microbiome because it requires specific sites for dehydroxylation. For example, the premise for *Eggerthella* sp. CAT-1 to cleave the C ring of catechins is to ensure that the B ring has a para-hydroxyl group, and the prerequisite for removing the para-hydroxyl group of the B ring is the cleavage of the C ring [137].

## 6. Bioavailability of Flavonoids

The chemical structure of flavonoids consists of an organic skeleton with a C6–C3–C6 aromatic ring, and the bioavailability of flavonoids is usually very low [120,138]. Structure, solute–solvent interactions, crystallinity, and thermodynamic properties are important determinants of the aqueous solubility of flavonoids [139]. The degree of methoxylation and glycosylation may also affect the solubility of these compounds; for example, the presence of glycone groups and unsubstituted hydroxyl groups increases polarity and thus aqueous solubility, whereas methoxylation generally decreases polarity. Furthermore, these compounds behave as weak acids (e.g., rutin has an overall pKa of 6.37 and contains four phenolic acid groups with pKa values ranging from 7.1 to 11.65). Therefore, the intestinal pH is insufficient to allow them to be completely dissolved. Because these are weak acids, their protonated forms are less soluble. Passive diffusion is also unlikely due to the low lipophilicity of the bound compound at intestinal pH [139]. In general, the bioavailability of hydrophobic flavonoids increases with the increase in glycosides, but their pharmacological activities may decrease [139]. Generally speaking, the bioavailability, metabolism, and bioactivity of flavonoids depend on their structural composition, number of hydroxyl groups, and the presence of structurally substitutable functional groups [140,141]. Previous studies have pointed out that the bioavailability of some flavonoids differs across various categories and grades, as follows: phenolic acids > isoflavones > flavonols > catechins > flavanones, proanthocyanidins > anthocyanins [142,143,144,145]. The bioavailability of different flavonoids has also been demonstrated in a study in which the minimum absorption of several flavonoids was assessed using urinary excretion (equivalent to a percentage of intake) as the primary indicator. The results showed that the minimum absorption of daidzein was 42.3 ± 3.0%, while the corresponding aglycone (daidzein) was only 27.5%. The minimum absorption of genistin was 15.6 ± 1.8%, while the corresponding aglycone (genistein) was 8.6%. In addition, the study also pointed out the urinary excretion of other flavonoids such as gallic acid (37.7 ± 1.0%), anthocyanidins (0.4 ± 0.3%), hesperidin (8.6 ± 4.0%), naringin (8.8 ± 3.17%), etc. [142]. However, it is worth noting that the detection of urinary excretion has a strong correlation with the intake dose of flavonoids, so these data may only have some reference value at present, and the specific differences need to be further explored. Glycosylation is a key modification of most flavonoids, and the modification of glycosidic substances by glycosylation enhances the bioactivity and bioavailability of natural products [146,147]. But interestingly, the absorption of flavonoid aglycones seems to be higher than that of their corresponding flavonoid glycosides to some extent, because aglycones can be absorbed from the small intestine, while most flavonoid glycosides need to be broken down by intestinal enzymes or intestinal flora before they can be directly absorbed [105]. These results, however, demonstrate that the bioavailability of some flavonoid glycosides appears to be higher than that of their corresponding aglycone counterparts [142]. Again, the absorption efficiency of quercetin glucoside is higher than that of its aglycone form [148], and the bioavailability of daidzin is higher than that of its corresponding aglycone [149]. These conclusions suggest that our research on the bioavailability of flavonoids may be more complicated than we initially imagined. In addition, factors such as diet, genetics, and metabolic diseases may also play a significant role in flavonoid bioavailability. The composition and activity of intestinal microorganisms may differ significantly between individuals, resulting in differences in the absorption, distribution, metabolism, and excretion of flavonoids, especially considering that dietary intake of flavonoids can promote more favorable composition of human intestinal microorganisms [150]. In addition, the food matrix will have a great impact on the bioavailability of flavonoids, and high-fat diets have been shown to change the structure and diversity of intestinal microorganisms [151]. For example, quercetin is lipophilic, and a high-fat diet can enhance its absorption [152]. Genetic polymorphisms of enzymes associated with flavonoid metabolism may also lead to inter-individual differences in bioavailability [153]. In addition, factors such as age, gender, race, and (pathological) physiological status may also affect the bioavailability of flavonoids [153]. Therefore, when facing special groups, the dosage and method of flavonoids need to be selected with great care [154]. Metabolic diseases are another factor affecting the bioavailability of flavonoids. In one study, it was found that intake of quercetin affects gut microorganisms and reduces the progression of atherosclerosis, but these effects are influenced by the presence of dietary plant polysaccharides and metabolic diseases [155]. In another study, 400 mg/kg of mangiferin was administered to diabetic and normal rats. The plasma area under the curve (AUC) of diabetic rats was significantly higher than that of the control group. In addition, the metabolic products of diabetic rats were also more than those of regular rats [156].

### 6.1. Formulation Modification

Modern formulation techniques have made it possible to significantly improve the therapeutic efficacy, release control, and bioavailability of drugs by preparing drugs or carriers at the nanoscale. Such technologies are often referred to as nanomedicine preparations or nanocarrier drug delivery systems. The combination of modern formulation techniques with flavonoids represents a promising area of research for improving their bioavailability [157,158]. Therefore, in this chapter, we mainly review nano-flavonoid drugs that improve pharmacokinetics, with the aim of illustrating the enormous potential of nanotechnology in enhancing the bioavailability of flavonoids. By preparing flavonoid compounds such as luteolin, naringenin, quercetin, etc. into corresponding nano-suspensions, solid lipid nanoparticles, liposomes, gel systems, micelles, and other nano-preparations, it becomes evident that the bioavailability can be significantly improved compared to the original drug [157,159,160,161,162,163] (Table 2).

In addition, there are several relatively novel multifunctional flavonoid nano-preparations, which are primarily used to meet personalized treatment needs and even visual treatment needs. For example, HA-RES-OPC-MMP NPs, a rhythmic flavonoid nano-preparation prepared in response to myocardial ischemia/reperfusion injury (rhythmic disease), has been shown to mitigate the damage during myocardial ischemia/reperfusion by targeting activated rhythm genes, as demonstrated in a pharmacodynamic study in mice [164]. In the case of L-EGCG-Mn, a flavonoid preparation that is both pH-responsive and MRI-imaging, the chelation of Mn^2+^ by EGCG is greatly reduced in acidic environments, which prompts its release for MRI capability [165]. These preparations, while improving the bioavailability of the original drug, can also be designed into highly effective nano-preparations with targeting, imaging, and even photothermal therapeutic effects, which are effective against a wide range of diseases including antioxidant, antitumor and, anti-inflammatory [166,167,168,169,170,171,172]. The nanoparticle-mediated flavonoid molecular targeting therapy strategy is another research direction with great potential. This technology is considered to overcome the limitations of traditional drugs, such as poor efficacy, insufficient specificity, and high toxicity [173]. The polymeric nanomicelles loaded with quercetin (P-gp inhibitor) and the estrogen receptor antagonist tamoxifen increased the oral bioavailability of quercetin by 2.9 times. Additionally, this formulation demonstrated superior in vivo antitumor efficacy [174]. Currently, a large number of nanoparticle formulations of various flavonoids have entered clinical stages, such as NCT02029352: Sinecatechins for the treatment of skin cancer, NCT03278925: Catechins for the treatment of liver cancer, and NCT01732393: Quercetin for the treatment of tumor inflammation. Of particular interest is the potential of EGCG in cancer therapy, with numerous clinical records already available, including NCT00917735, NCT02580279, and NCT02891538 [175].

**Table 2:** Nano preparation for improving bioavailability of flavonoids.

Type	Substance	Name	Administration	Dose	Animal/Cell Line	Disease	Pharmacokinetics (Compared with Pure Substances)	Ref.
Nanosuspensions	Baicalin	BG-NS and BG-MS	Oral administration	85, 170, 340 and 680 mg/kg	Wistar rats	---	AUC_(0–t)_ increased by about 2.22 times and 1.37 times respectively; Vz/F reduced by about 2.27 times and 1.28 times respectively…	[176]
	Luteolin	SLNC	Oral gavage	20 mg/kg	Male Sprague Dawley rats	---	Bioavailability increased by about 3.48 times	[177]
	Daidzein	F-A and F-B	Co-incubation	50–400 μM	RG2	Brain glioma	---	[178]
	Naringenin	TPNS	Oral administration	30 mg/kg	Male Sprague Dawley rats	---	C_max_ increased by about 2.1 times, AUMC_0–∞_ increased by about 3.76 times…	[179]
	Silybin	SPCs-NPs	Oral gavage	50 mg/kg	Male Sprague Dawley rats	Liver protection	AUC_0–∞_ increased by about 124.70 times; CL reduced by about 124.58 times; T_max_ increased by about 8.82 times…	[180]
	Kaempferol	TPGS-KAE-NSps	Oral gavage and vein injection	15 mg/kg, ig; 5 mg/kg, iv	Female Balb/c mice	Breast cancer	C_max_ increased by about 2.41 times; AUC_0–t_ increased by about 4.83 times, T_1/2_ increased by about 2.64 times.	[181]
	Hydroxy genkwanin	HGK-NSps	vein injection	10, 20, 40 mg/kg	Female NU/NU nude mice	Breast cancer	---	[182]
	Naringenin	NRG-NS	Oral administration	20 mg/kg	Female Wistar rats	---	C_max_ increased by about 2 times; AUC_0–24 h_ increased by about 1.8 times;	[183]
	Baicalin	BCA-NS/NCCS	Oral administration	10 mg/mL	Male Wister rats	---	AUC_(0–24)_ increased by 1.85 times; C_max_ increased by about 1.90 times…	[184]
Liposomes	Quercetin	Quercetin liposomes	Co-incubation	---	RBL-2H3	Allergic	Anti-allergic activity is higher than that of raw drug	[185]
	Fisetin	---	Intraperitoneal injection	21 mg/kg	C57BL/6J mice	Lung cancer	Relative bioavailability increased by about 47 times	[186]
	Rutin	MP-LR	Oral administration	16.15 mg	C57 BL/6N mice	Obesity	The RQ-AUC value (the lower the ratio indicates better fatty acid metabolism) is significantly lower than the raw material drug (*p* < 0.05)	[187]
	Rutin	RGD-RUT-LIPO and ABX-RUT-LIPO	Tail vein injection	5 mg/kg (Rutin equivalent)	Male Sprague Dawley rats	Thrombus	Shortened clotting time; Relative bioavailability increased by about 3 times.	[188]
	Licochalcone A	LCA-Liposomes	Oral administration	30, 60 mg/kg	Male Sprague Dawley rats	Renal injury	AUC_0–24_ increased by about 2.86 times; C_max_ increased by about 2.49 times; MRT_0–t_ increased by about 1.26 times;	[189]
Solid lipid nanoparticles	Morin hydrate	MSN	Oral gavage	50 mg/kg	Male Sprague Dawley rats	Cervical cancer	C_max_ increased by about 2.95 times; AUC increased by about 3.10 times;MRT increased by about 2.04 times;	[190]
	Hydroxysafflor yellow A	HSYA SLN	Oral administration	20 mg/kg	Male Sprague Dawley rats	Nerve injury	C_max_ increased by about 7.76 times; AUC increased by about 3.99 times; Oral absorption in rats increased by about 3.97 times.	[191]
	Baicalin	OX26-PEG-CSLN	vein injection	4.42 mg/kg	Male Sprague Dawley rats	Cerebral ischemia reperfusion injury	AUC increased by about 5.69 times; C_max_ increased by about 6.84 times;	[192]
	Naringenin	Nrg-SLNs	Oral administration	6 mg/mL	Wistar rats	inflammation; Antioxidant	AUC_0→∞_ increased by about 17.44 times; MRT_0→∞_ increased by about 8.81 times; The overall bioavailability is increased by about 12 times;	[193]
	Naringenin	NRG-SLNs	intratracheal instillation	20 mg/kg	Male Sprague Dawley rats	---	C_max_ increased by about 1.62 times; AUC_0→∞_ increased by about 3.66 times; MRT increased by about 3.33 times; Relative bioavailability increased by about 2.53 times;	[194]
	Puerarin	Pue-SLN	Oral gavage	20 mg/kg	Sprague Dawley rats	---	AUC_0→∞_ increased by about 3.00 times; MRT increased by about 1.79 times; CL reduced by about 3.21 times…	[195]
Nanoemulsions	Breviscapine	---	Oral administration	---	Male Wister rats	---	C_max_ increased by about 2.87 times; AUC_(0–t)_ ncreased by about 2.57 times; Relative bioavailability reached 249.70%;	[196]
	Baicalein	BCL-NEs	Oral gavage	25 mg/kg	Sprague Dawley rats	---	AUC_0–t_ increased by about 5.25 times; C_max_ increased by about 7.66 times; Relative bioavailability reached 524.7%	[197]
	Acetylpuerarin	---	Oral administration	30 mg/kg	Wistar rats	Cerebral ischemic reperfusion injury	C_max_ increased by about 2.89 times; AUC_0–t_ increased by about 2.57 times;	[198]
	Luteolin	NECh-LUT	intranasal administration	32 μg/kg	Male Wistar rats	Neuroblastoma	AUC_0-∞_increased by about 4.40 times; T_1/2_ increased by about 10 times;	[199]
	Fisetin	---	intraperitoneal injection	112.5 mg/kg	C57BL/6J mice	Lung cancer	Relative bioavailability increased by about 24 times	[200]
	Isoliquiritigenin	ISL-NE	Ocular instillation	50 μL 0.2% (*w*/*v*)	Male New Zealand white rabbits	Corneal neovascularizaion	C_max_ of tear, cornea, Conjunctiva Aqueous humor increased by about 8.70, 3.95, 298.75 and 1.88 times respectively; AUC_0–8 h_ increased by about 5.76, 7.80, 356.57 and 2.13 times respectively…	[201]
Hydrogel	Catechin	CA-NG4	Transdermal administration	5 mg (CA equivalent)	male Wistar rats	Antioxidant	AUC_0–∞_increased by about 10.33 times; Relative bioavailability increased to 894.73%	[202]
Metal-organic frameworks	Baicalin	PEG-FA@ZIF-8@BAN	vein injection	---	female BALB/c mice	Breast cancer	Stronger tumor suppressor effect.	[203]
Nanoparticles	Epigallocatechin gallate	CE-HK NP	Intratumoral injection	20 mg/kg and 40 mg/kg	Male BALB/c Nude	Liver cancer	Tumor suppression effect increased by about 2.77 times.	[204]
Metal nanoparticles	Hesperetin	Au-mPEG_(5000)-_S-HP NPs	Intraperitoneal injection(IP)	1.5 mg/0.5 mL	Male, Wistar strain albino rats	Liver cancer	---	[205]
Magnetic nanoparticles	Quercetin	Fe_3_O_4_@PCA-PEG-FA	Co-incubation	50, 100, 200 μg/mL	MDA-MB-231 and HeLa	---	---	[206]
NA	Breviscapine	BVP-NS, BVP-LP, BVP-PLC	Oral gavage	20 mg/kg	Male Sprague-Dawley rats	---	The relative bioavailability increased to 245.97%, 237.51%, and 471.32, respectively;	[207]
Nanogel	Breviscapine	BRE-NG	intranasal administration	3, 10, 50, 100 mg/mL	Male Sprague-Dawley rats	Cerebral ischemia reperfusion injury	Absolute bioavailability increased by about 142.80 times	[208]

### 6.2. Structural Modification

Structural modification of flavonoids is an important approach to promote their development in the field of medicine. Modern research shows that altering the skeleton structure of flavonoids has a significant impact on their absorption, metabolism, and distribution. Common modification methods include chemical modification, microbial methods, and enzymatic methods [209]. Generally speaking, chemical modification is the most direct and simple modification method, while the other two methods are more commonly used in large-scale industrial production. Chemical modification aims to replace selected chemical parts with other functional groups to achieve more desirable properties. As a key strategy to improve the bioavailability of natural products or drugs, improve the pharmacological effects and pharmacokinetic properties, it has broad application prospects. By performing various chemical modifications on flavonoids, their solubility, stability, and bioactivity can be optimized, thereby enhancing the efficacy of these compounds and expanding their range of applications.

#### 6.2.1. Acetylation

The bioavailability of flavonoids is closely related to their lipophilicity, and the methylene groups in acetamide derivatives result in increased lipophilicity of the compounds, thus improving their bioavailability. Isika et al. [210] converted all hydroxyl groups in the structures of quercetin, apigenin, and luteolin into acetamide groups and evaluated the bioavailability of both original substances and their acetylated derivatives (quercetin tetra acetamide, apigenin di acetamide, and luteolin tri acetamide) in vitro. The results showed that acetylated flavonoids had higher bioavailability compared with unmodified compounds. Of course, this could all be attributed to the presence of methylene and amide groups [211]. In addition, the researchers synthesized acetylated arbutin [212], epigallocatechin-3-gallate [213], and acylated puerarin (puerarin esters) [214], in order to improve the lipid solubility of the drugs. In particular, the pharmacokinetics of acylated puerarin were investigated in SD rats. The results clearly showed that esters can improve the bioavailability of the original drug puerarin [215]. This is because enhanced lipid solubility allows these compounds to more easily penetrate the lipid bilayer of the cell membrane [216]. However, it is still important to note that acetylated flavonoids may exhibit certain changes in bioactivity. For example, acetylated flavonoid glycosides reduce the original activity against diet-induced obesity (DIO) and hepatic steatosis [217].

#### 6.2.2. Glycosylation

Most natural flavonoids exist in the form of glycosides. Aglycones (flavonoids) are combined with glycone groups through glycosidic bonds to form flavonoid glycosides. Most flavonoid glycosides are metabolized in the body into corresponding aglycones with smaller analytical structures before they are absorbed. Therefore, some opinions think that the bioavailability of flavonoid aglycones is always higher than that of their corresponding glycosides. However, a previous study found that the absorption efficiency of quercetin glucoside was higher than that of its aglycone form [148]. Other studies also have reported that glucosylation may increase the bioavailability of flavonoids such as anthocyanins [218]. Of course, the impact of glycosylation on the bioavailability of flavonoids cannot be determined solely based on individual studies. The type, amount and nature of glycosidic bond may all have a greater impact on the bioavailability of flavonoids [219].

#### 6.2.3. Methyl Etherification

Flavonoids such as EGCG have multiple phenolic hydroxyl groups. A large number of active phenolic hydroxyl groups contribute to their drug activity but are also the culprits for their reduced bioavailability [220]. For the structural modification of EGCG, the methyl etherification pathway was used to achieve higher bioavailability by converting some or all of the eight active phenolic hydroxyl groups in the A, B, and D rings into methyl ether derivatives [221,222,223]. It is worth noting that methyl etherification may not only have beneficial effects on the human body but also play a certain role in protecting plants against pests and diseases. For example, methyl etherified naringenin has been found to be used to fight fungal pathogens that can infect corn [224].

#### 6.2.4. Esterification

Esterification of the carboxyl groups of flavonoids to increase their lipid solubility and enhance their affinity for cell membranes is an effective method to improve the bioavailability of flavonoids. The researchers prepared a series of baicalin ester derivatives by adding fatty alcohols to baicalin in a non-aqueous medium for the esterification reaction. Interestingly, the esterified baicalin not only exhibits stronger antibacterial activity, but also increase the lipophilicity of the original drug. Of course, the researchers also pointed out that fatty alcohol chains that are too long may cause excessive lipophilic activity and form extracellular aggregates, thereby reducing their activity [225]. In addition, the researchers also synthesized baicalin ester derivatives with different fatty acid chain lengths through a whole-cell catalytic esterification reaction and evaluated the absorption of baicalin esters compared to baicalin using a Caco-2 cell model. The results showed that the absorption efficiency of baicalin ester was much greater than that of baicalin, with a maximum difference of up to 10 times [226].

#### 6.2.5. Acylation

Due to the poor stability and low solubility of some flavonoids in lipids, acylation modification has become the main means to improve their bioactivity. Compared with unacylated flavonoids, acylated flavonoids have greatly improved their activity. Phloridzin docosahexaenoate (PZ-DHA) is an omega-3 fatty acid ester of a flavonoid precursor. Recent research has shown that PZ-DHA, in contrast to pure Phloridzin, demonstrates significant antitumor metastatic activity. This is achieved through the inhibition of angiogenesis both in vitro and in vivo, as well as by reducing the proliferation and migration of human umbilical vein endothelial cells (HUVECs) (*p* < 0.01) and human microvascular venous endothelial cells (HMVECs) (*p* < 0.05) [227]. Moreover, PZ-DHA currently demonstrates significant advantages in the treatment of skin cancer, triple-negative breast cancer, overcoming tumor resistance in triple-negative breast cancer, and combating leukemia [227,228,229,230]. In conclusion, acylation increases the lipophilicity of flavonoids, making them more easily absorbed by cells, and acylated flavonoids have demonstrated significantly greater pharmacological activity in antiviral, antioxidant, anti-inflammatory, antimicrobial, anticancer and other. However, some studies have pointed out that high doses of acylated quercitrin, compared to its non-acylated form, may even exacerbate cellular oxidative stress. This suggests that precise dosage control is essential, especially when considering flavonoid compounds modified by acylation [231].

## 7. Prospect

Despite showing significant bioactivity in vitro, the bioavailability of flavonoids in vivo remains a major bottleneck that limits their widespread application [232]. To address this issue, future research may explore various approaches, including optimizing drug delivery technologies, chemically modifying flavonoid molecules, and examining the influence of gut microbiome, among others.

With advances in nanotechnology, delivery systems for flavonoids are undergoing rapid innovation. Techniques such as nanoparticles, liposomes, and solid dispersions have significantly enhanced their bioavailability [233]. Research indicates that carriers like nanoparticles and liposomes can effectively protect flavonoids from degradation in the digestive system and enhance their therapeutic efficacy through targeted delivery mechanisms [234]. Furthermore, the chemical modification of flavonoid compounds is a significant strategy for enhancing their bioavailability. Structural alterations, such as introducing new functional groups to the flavonoid molecule, can improve solubility, stability, and lipophilicity, thereby facilitating absorption and distribution [235]. Future integration of efficient molecular design and synthesis methods may significantly enhance the bioavailability of flavonoid compounds. Moreover, recent studies have shown that the gut microbiome plays a crucial role in the metabolism of flavonoid compounds. The gut microbiome not only generates bioactive metabolites from flavonoid metabolism but also influences their absorption and biological effects within the host organism [127]. This finding offers new insights into personalized nutrition and the application of flavonoid compounds. Future research could explore the functions of different gut microbiome communities to further investigate their interactions with flavonoid compounds and develop personalized health management strategies based on microbiome-drug interactions [236]. For example, the probiotic strain Bifidobacterium pseudocatenulatum B7003 not only provides benefits for gut transit but also enhances the antioxidant activity and bioavailability of flavonoids by converting flavonoid glycosides in dairy-like products into their aglycone form [237]. It is worth noting that flavonoids are also considered a nutritional source for probiotics by providing energy and nutrients to promote the growth and reproduction of probiotics [238,239]. The addition of banana peel polyphenol extract to yogurt enhanced the vitality of B. lactis and L. acidophilus strains [240]. Undoubtedly, this strategy holds significant growth potential, as the appeal of a win–win situation is difficult to overlook. The strategy also has been implemented by Shehata et al., who developed functional beverages from taro leaf extract (TLE) and probiotics. This study highlighted that the polyphenols (including flavonoids) in these extracts play a significant role in enhancing the viability and stability of probiotics. Research has demonstrated that functional beverages are capable of maintaining probiotic concentrations exceeding 7.00 log cfu/mL after 30 days of storage, thereby supporting essential health-promoting functions. Additionally, the presence of probiotics has been shown to enhance the antioxidant capacity of polyphenolic compounds [241]. In the future, advancements in gut health, immune function, cardiovascular health, and so on, may all be positively influenced by ongoing research into the combination of flavonoids and probiotics [242,243,244,245].

## 8. Conclusions

This review comprehensively explores the multifaceted nature of botanical flavonoids, emphasizing their diverse bioactivities and the challenges associated with their low bioavailability. We highlight the critical role of gut microbiome in the metabolism and bioactivation of flavonoids, revealing how microbial modifications can enhance their therapeutic potential. Furthermore, it delves into innovative strategies such as nano-formulations and chemical modifications (e.g., acylation, glycosylation) to improve the bioavailability and bioactivity of flavonoids. These approaches offer new avenues for optimizing the pharmacokinetics and therapeutic efficacy of these natural compounds. We underscore the need for future research to focus on advanced delivery systems and structural modifications, aiming to translate the promising in vitro findings of flavonoids into clinical and health applications. By addressing the bioavailability bottleneck and leveraging the interactions between flavonoids and the gut microbiome, this work paves the way for personalized nutrition and therapeutic strategies, potentially impacting gut health, immune function, and cardiovascular health.

## Figures and Tables

**Table 1:** Classification, chemical characterization, common biological sources, representatives, and applications of flavonoids.

Subtype	Structure Backbone	Chemical Characterization	Common Biological Sources	Representatives	Applications	Ref.
Flavone	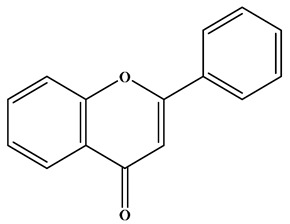	There is a double bond between the C2 and C3 positions; a ketone group is at the C4 position; and the C2 position is connected to the B ring.	Celery, tea, red peppers, and oranges	Apigenin; Luteolin	Cancer, cardiovascular disease, neuroinflammation inflammation, anti-diabetic, antibacterial, antioxidant, and antiviral, etc.	[26,27,28]
Isoflavone	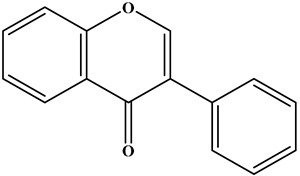	There is a double bond between the C2 and C3 positions; a ketone group is at the C4 position; and the C3 position is connected to the B ring.	Soybeans, and soy-derived products	Genistein; Daidzein	Antioxidant, diarrhea relief, procoagulant activity, and anticancer, etc.	[29,30,31]
Flavonol	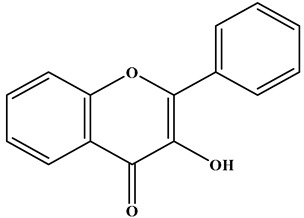	There is a double bond between the C2 and C3 positions; a ketone group is at the C4 position; a hydroxyl group is connected to the C3 position; and the C2 position is connected to the B ring.	Apples, cherries, plums, apricots, berries, onions, kale, and leeks	Quercetin; Myricetin; Kaempferol	Cardiovascular diseases, anticancer, antioxidation, and neuroprotection, etc.	[32,33,34,35]
Flavanol	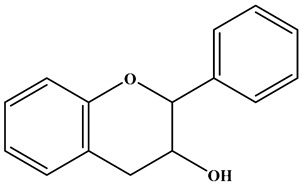	There is no double bond between the C2 and C3 positions; there is no ketone group at the C4 position; a hydroxyl group is connected to the C3 position; the C2 position is connected to the B ring.	Broccoli, onions, asparagus, apples, and tea	Epicatechin; Epigallocatechin	Antioxidant, anticancer, and anti-inflammatory, etc.	[36,37,38]
Flavanone	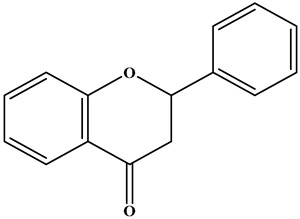	There is no double bond between the C2 and C3 positions; there is a ketone group at C4 position; and the C2 position is connected to the B ring.	Citrus Fruits	Hesperidin; Naringin; Paclitaxel	Anticancer, anti-inflammatory, antibacterial, antioxidant, antiviral, and lipid-lowering, etc.	[2,39,40,41]
Chalcone	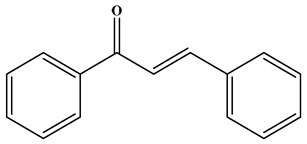	No C ring (open-chain flavonoids).	Leguminosae, Moraceae, Zingiberaceae, and Cannabaceae	Xanthohumol; Corylifolinin	Antioxidant, antibacterial, anti-inflammatory, antiviral, and anticancer, etc.	[42,43,44]
Anthocyanidin	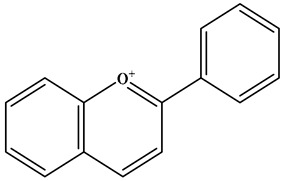	2-phenylbenzopyranyl cation structure; there is a double bond between the C1 and C2; there is a double bond between the C3 and C4. The C2 position is connected to the B ring.	Blueberries, red cabbage, tomatoes, purple sweet potatoes, and eggplant	Delphinidin; Cyanidin; Petunidin; Peonidin; Malvidin; Pelargonidin	Eye health, cardiovascular disease, antiobesity, antidiabetes, antibacterial, anticancer activity and neurodegenerative diseases, etc.	[25,45,46]

## Data Availability

No data was used for the research described in the article.

## Abbreviations

AKT: Protein kinase B; AP1: Activator protein 1; BCRP: Breast cancer resistance protein; CAT: Catalase; CBG: Cytosolic β-glucosidase; Cd: Cadmium; c-MET: Cellular-mesenchymal epithelial transition factor; C-Myc: Cellular Myelocytomatosis oncogene Gene; cGAS-STING: Cyclic GMP-AMP synthase-stimulator of interferon genes; COX-2: Cyclooxygenase-2; csgA/B: Curli subunits A/B; DIO: Diet-induced obesity; EGCG: Epigallocatechin gallate; EGFR: Epidermal growth factor receptor; EHEC: *Escherichia coli;* EpRE: Electrophile-responsive element; ERK1/2: extracellular regulated protein kinases1/2; FLR: flavonoid reductase; GLUT-2: Glucose transporter 2; GSH-Px: Glutathione peroxidase; HO-1: Heme oxygenase 1 LDL: Low-density lipoprotein; IL-6: Interleukin-6; IL-1β: Interleukin-1β; IL-17: Interleukin-17; IKK: IκB kinase; iNOS: Inducible nitric oxide synthase; LPH: Lactase phlorizin hydrolase; MAPK/Erk: Mitogen-activated protein kinase/Extracellular regulated protein kinases; MITF: Microphthalmia transcription factor; NADH: Nicotinamide adenine dinucleotide; NADPH: Nicotinamide adenine dinucleotide phosphate; NF-κB: Nuclear factor κB; NOTCH: Notch Receptor; NOX: Nicotinamide adenine dinucleotide phosphate oxidase; Nrf2: Nuclear factor erythroid 2-related factor 2; PDGF: Platelet-derived growth factor; Perk: Phospho-ERK; PGE2: Prostaglandin E2; PLC/PKC: Phosphoinositide-specific Phospholipase C/Protein kinase C; PI3K: Phosphoinositide 3-Kinase; pJNK: Phospho c-Jun N-terminal kinase; P-P38: Phospho P38 mitogen-activated protein kinase; Rac1: Ras-related C3 botulinum toxin substrate 1; Raf: Rapidly Accelerated Fibrosarcoma; Ras: Rat Sarcoma; RNS: Reactive nitrogen species; ROS: Reactive oxygen species; SOD: Superoxide dismutase; SGLT-1: Sodium-dependent glucose transporter 1; TAX: Taxifolin; TF3: Theaflavin-3,3′-digallate; TLR: Toll-like receptors; TNF-α: Tumour necrosis factor-α; VEGF: Vascular Endothelial Growth Factors; YAP/TEAD: Yes-associated protein/Transcriptional Enhanced Associate Domains.
